# The time course of neuromuscular impairment during short‐term disuse in young women

**DOI:** 10.14814/phy2.14677

**Published:** 2021-01-11

**Authors:** Rob J. MacLennan, David Ogilvie, John McDorman, Ernest Vargas, Arielle R. Grusky, Youngdeok Kim, Jeanette M. Garcia, Matt S. Stock

**Affiliations:** ^1^ Applied Neuromuscular Physiology Laboratory Oklahoma State University Stillwater OK USA; ^2^ School of Kinesiology & Physical Therapy University of Central Florida Orlando FL USA; ^3^ Neuromuscular Plasticity Laboratory Institute of Exercise Physiology and Rehabilitation Science University of Central Florida Orlando FL USA; ^4^ Department of Kinesiology and Health Sciences Virginia Commonwealth University Richmond VA USA; ^5^ Department of Health Sciences University of Central Florida Orlando FL USA

**Keywords:** disuse, motor unit, muscle, voluntary activation, weakness

## Abstract

Skeletal muscle disuse results in rapid functional declines. Previous studies have typically been at least 1 week in duration and focused on the responsiveness of men. Herein, we report the timeline of initial impairments in strength, voluntary activation (VA), and motor unit control during 2 weeks of knee joint immobilization. Thirteen women (mean age =21 years) underwent 2 weeks of left knee joint immobilization via ambulation on crutches and use of a brace. Participants visited the laboratory for testing on seven occasions (two familiarization visits, pretest, 48 and 72 h, 1 and 2 weeks). Knee extensor isometric and concentric isokinetic strength at two velocities (180 and 360 degrees⋅s^−1^), VA, and submaximal vastus lateralis motor unit activity were evaluated. Moderate‐to‐large decreases in isometric and concentric strength at 180 degrees⋅s^−1^ and VA were observed within 48 hours. Isometric strength continued to decline beyond 72 h, whereas other variables plateaued. The *B*‐term of the motor unit mean firing rate versus action potential amplitude relationship demonstrated a moderate increase 1 week into immobilization, suggesting that greater firing rates were necessary to maintain pretest torque levels. Concentric strength at a velocity of 360 degrees s^−1^ was not affected. Decreases in knee extensor strength occur within a matter of days after immobilization, although the time course and magnitude vary among assessment methods. These changes are mediated by the nervous system's capacity to activate skeletal muscle. Clinically appropriate interventions which target nervous system plasticity should be implemented early to minimize the rapid functional impairments associated with disuse.

## Graphical abstract

The need to immobilize the knee joint is common following injury and surgery. Our findings demonstrate rapid and large decreases in maximal strength and neuromuscular function (voluntary activation and motor unit behavior) within 1 week of knee joint immobilization in young women. For clinicians, these findings highlight the need for early interventions that may mitigate immobilization‐induced declines in muscle function.

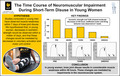

**Key Points Summary**

- Muscle disuse studies have typically been ≥1 week in duration and focused on the responsiveness of men.
- We examined the precise time course of neuromuscular impairment during 2 weeks of knee joint immobilization in young women.
- Maximal strength and voluntary activation showed moderate decreases within 48 hours, whereas motor unit control demonstrated signs of inefficiency at 1 week.
- No evidence of muscle weakness was observed when tested at a rapid isokinetic velocity, highlighting the importance of proper test selection in clinical settings.
- Impairments in the central nervous system's ability to activate and control skeletal muscle occur more rapidly than previously reported.

## 1 INTRODUCTION

Physiological systems exhibit a substantial degree of plasticity in response to imposed physical demands. Whereas increased external loading results in several benefits to the musculoskeletal system, disuse causes dramatic declines in muscle strength, atrophy, cardiorespiratory impairments, and decreases in bone mineral density (Campbell et al., 2019). These changes are commonly observed during bed rest (Reidy et al., 2017) and single‐limb injury with subsequent immobilization lasting between 7 days up to several weeks (Tesch et al., 2016). While declines in physical function are reversible with rehabilitation (Hvid et al., 2010), all adults are susceptible to disuse‐related muscular impairments, regardless of age (Hvid et al., 2018) and initial strength levels (Deschenes et al., 2017).

Changes in both muscle strength and mass have been commonly assessed in disuse studies. The literature generally indicates that the rate at which strength is lost is greater than the rate of muscle mass loss (Cook et al., 2014; Deschenes et al., 2002, 2017; Reidy et al., 2017). As the decrease in muscle strength cannot be fully explained by muscle atrophy, factors at or proximal to the neuromuscular junction have received some attention in the literature. Indeed, decreases in voluntary activation (VA) (Clark et al., 2014; Cook et al., 2014) and myoelectric activity (Deschenes et al., 2002, 2009), among other variables, have been commonly observed in limb immobilization studies. Clark et al. (2014) used transcranial magnetic stimulation over the motor cortex to report a strong relationship between the loss of isometric wrist flexor strength and increased corticospinal inhibition. Further support for the importance of neural, rather than muscular, impairments during disuse comes from some studies which have demonstrated a significant loss in voluntary strength with little change in resting twitch force, suggesting maintenance of contractile function (Cook et al., 2014; Seynnes et al., 2010). However, to our knowledge, a clear analysis of the time course by which these changes took place has not been carried out, particularly in the early phases of immobilization. Furthermore, changes in motor unit recruitment and firing rates during disuse have yet to be investigated.

Within the last decade, the ability to decompose surface EMG signals, obtained during voluntary contractions, into their constituent motor unit action potential trains has provided investigators with a more comprehensive understanding of motor unit behavior (Nawab et al., 2010; Negro et al., 2016). Given that the output of surface EMG signal decomposition techniques often provides firings from 20 to 50 motor units for a single contraction, investigators have commonly used regression‐based statistical approaches (i.e., analysis of slopes and intercepts) to facilitate data interpretation. These techniques have been used to study the motor unit adaptations that occur in response to aging (Mota et al., 2020), resistance training (Pope et al., 2016), and fatigue (Stock & Mota, 2017), in addition to exploration of gender differences in neuromuscular function (Parra et al., 2020).

In our view, a thorough review of the current limb immobilization literature shows two clear gaps in our understanding. First, most investigators have focused their attention on the responsiveness of men, with some studies including both men and women (Cook et al., 2010; Deschenes et al., 2002; Vaughan, 1989). Furthermore, compared to the knee joint, a greater proportion of women have been included in upper‐limb immobilization studies (Campbell et al., 2019). One study by Deschenes, McCoy, Holdren, et al. (2009) reported that 7 days of knee joint immobilization resulted in decreased knee extensor strength and neuromuscular function in both genders, but the declines were more pronounced for women. Given that young women participating in sporting events experience knee injuries at a rate substantially greater than that for men (Renstrom et al., 2008), this is a critical area of study. As highlighted in the review by Campbell et al. (2019), we have a limited understanding of the gender‐specific attributes of knee joint dysfunction during periods of immobilization lasting less than 1 week. Second, while the majority of study durations have been between 7 (Deschenes, McCoy, Holdren, et al., 2009; Deschenes et al., 2017) and 35 days (Tesch et al., 2004), limited data concerning short‐term limb immobilization exist. An intriguing aspect of this fact is that the overall percent decline in strength has been similar among studies longer than 7 days (Campbell et al., 2019), suggesting that critical mechanistic changes may be occurring within only a few days of limb immobilization. Overall, there is a paucity of data concerning short‐term knee joint immobilization in women, thus, hindering the ability of clinicians to prescribe evidence‐based treatments following injury or surgery.

Given the significant gaps in the literature, the purpose of the present study was to examine the precise time course of muscle strength loss and neuromuscular impairments during short‐term knee joint immobilization in women. Extrapolating from previous investigations, we hypothesized that significant decreases in muscle strength would be observed within only a few days of knee joint immobilization. We further hypothesized that decreases in voluntary activation and altered motor unit behavior during submaximal contractions would be found within 1 week of immobilization. Specifically, when testing the same absolute torque level throughout the study, we speculated that vastus lateralis motor units would need to fire at higher rates, be recruited at lower torque levels, and additional high‐threshold motor units would need to be recruited. Each of these adaptations would be viewed as compensatory adjustments in neuromuscular control that would offset diminished motor unit twitch torque in response to disuse.

## 2 METHODS

### 2.1 Overview of the study design

Participants visited our laboratory seven times over about a 3‐week period. The first two visits were for extensive familiarization with the testing protocol. The following five visits occurred before immobilization (PRE), as well as 48 hours (48HR), 72 h (72HR), 1‐week (1 Week), and 2‐weeks (2 Weeks) later. These visits were strictly scheduled to be at the same time of day (±1 h). At the end of PRE, participants were fitted with a left knee brace and crutches. Laboratory tests, described below in the order in which they were performed, included submaximal contractions for vastus lateralis motor unit analyses, isometric maximal voluntary contractions (MVC), percent VA, and concentric isokinetic strength at two velocities (180°⋅s^−1^ and 360°⋅s^−1^). Due to the potential that excessive testing may provide a training stimulus, the number of voluntary contractions performed during each testing session was kept to a minimum. Specifically, excluding the submaximal warm‐up, each participant performed only two isometric contractions at 50% of the PRE torque level, two MVCs, and two maximal concentric muscle actions at each velocity (i.e., two submaximal and six maximal contractions during each testing session). During the 2‐week immobilization period, the participants avoided alcohol, kept their dietary and caffeine habits consistent, and refrained from bearing weight on their left leg.

### 2.2 Participants

Twenty‐six college‐aged women enrolled in the study. Participants were recruited with flyers, social media, laboratory and university websites, and presentations in classrooms and club meetings. Inclusion criteria included identifying as a woman, being between the ages of 18 and 35 years, and having a body mass index ≤30 kg/m^2^. Exclusion criteria included those with a personal or family history of blood clots, neuromuscular or metabolic disease, osteoarthritis, surgery on the hip or knee joints, use of an assistive walking device within the previous year, myocardial infarction within the past year, current pregnancy, use of contraceptives within the previous 90 days, musculoskeletal pain or discomfort in any of the major joints, and obesity (body mass index >30 kg/m^2^). Prior to enrollment, participants completed an in‐house health questionnaire and a Physical Activity Readiness Questionnaire + (PAR‐Q +) to determine eligibility. All participants read, understood, and signed an informed consent form. The study protocol was approved by the University of Central Florida's Institutional Review Board (Study # BIO‐17–13642). Participants were compensated $350 for completing the study.

Of the 26 participants who enrolled, 11 withdrew. Reasons for withdrawing included, but were not limited to, time constraints, changes in contraceptive use, difficulty accomplishing daily activities and subsequent inability to comply with the study protocol, and discomfort. Of the 15 participants who completed the study, two were removed from this manuscript's statistical analysis, one because of accelerometer data demonstrating non‐compliance and another due to consistently low motor unit yields (details described below). All participants included in the final dataset were right leg dominant (based on kicking preference) and, therefore, our analyses focused on immobilization of the non‐dominant limb.

### 2.3 Immobilization procedures

At the end of PRE, participants were properly fitted with a knee joint immobilization brace (T Scope® Premier Post‐Op Knee Brace, Breg, Inc., Carlsbad, CA, USA). The brace was locked at 90° of flexion, which raised the foot off of the ground and prevented normal weight‐bearing, allowing the knee extensors to stay relaxed. The brace was only removed in bed as to not disrupt sleep and to allow for cleaning of the skin. During bathing, participants were asked to continue wearing the brace and to keep it dry by covering it with a large plastic bag. For their safety and comfort, each participant was offered a shower chair (Medline Shower Chair Bath Seat with Padded Armrests and Back, Medline Industries, Inc., Northfield, IL, USA). For ambulation, participants were provided with and fit for axillary crutches (Cardinal Health Axillary Crutch, Adult, Height 62–70 in, Adjustable, Cardinal Health, Inc., Dublin, OH, USA). Participants were trained in the proper use of crutches including navigation of curbs, stairs, and ramps.

Participants wore a stocking (Rolyan Extra Soft Stockinette, 100% Cotton, Performance Health, Warrenville, IL, USA) between the brace and their skin that was measured to extend from the proximal thigh to the ankle. The purpose of this stocking was to provide comfort and minimize the risk of skin irritation. This stocking was worn during the daytime, with the brace, and was only removed for sleeping. A compression stocking was also provided that was worn while sleeping (Medi‐Pak Anti‐Embolism Stockings, McKesson, San Francisco, CA, USA). The purpose of this stocking was to reduce the risk of blood clots. Participants were encouraged to maintain basic hygiene and were advised to notify their point of contact on the study team immediately if they experienced any adverse skin reactions or swelling.

Consistent with previous knee joint immobilization studies (Deschenes et al., 2008; Deschenes, McCoy, Holdren, et al., 2009), each participant performed light range of motion movements at the ankle and knee while lying supine in bed. These activities were performed twice daily (morning and evening) to minimize the risk of vascular pathology and muscular contractures. A video containing instructions was provided to participants, as was as a handout with detailed directions of performance of the exercises. The compressive stocking was worn during the exercise. Finally, to further ensure compliance and safety, study investigators text messaged or spoke with the participants via telephone daily.

### 2.4 Assessment of isometric and concentric torque

All of the muscle strength measurements described in this study were performed with the left knee extensors via a Biodex System 4 isokinetic dynamometer (Biodex Medical Systems, Shirley, NY, USA). The participant's axis around the knee was aligned with the axis of rotation of the dynamometer. Each participant's chair settings were recorded during the first familiarization visit and used during every subsequent testing session. The chair was adjusted so that isometric torque testing was performed at hip and knee joint angles of 110°. The participants were secured in the chair by straps over their hips, chest, and right leg to reduce unnecessary movement. The lower leg was secured to an Anti‐Shear attachment (Biodex Medical Systems, Shirley, NY, USA), with the pad over the tibialis anterior muscle and just superior to the malleoli. During all contractions, participants grasped the stabilization handles at their sides. Prior to testing, participants performed a warm‐up consisting of a single 10‐second contraction at 50% of their perceived maximal torque.

### 2.5 Vastus lateralis motor unit behavior

Following a submaximal warm‐up at the PRE visit (i.e., prior to immobilization), each participant performed three, 5‐second MVCs separated by 3 minutes of rest. The highest torque from the three MVCs was used to standardize the submaximal testing among the participants. Following determination of the MVC, participants performed trapezoidal isometric contractions by tracing visual templates on a computer monitor. Each participant increased isometric torque from 0 to 50% MVC in 5 seconds (10%/second), held 50% MVC constant for 15 s, and decreased isometric torque from 50 to 0% MVC in 5 s (10%/second). Two submaximal contractions were performed, each separated by a 3‐minute rest period. The participants were instructed to maintain their torque output as close as possible to the visual template. Each participant's absolute PRE 50% MVC torque level was used throughout the duration of the study. In other words, if maximal strength declined during the immobilization period, a greater relative percentage of isometric torque was required to achieve the same absolute output.

Bipolar surface EMG signals were recorded from the vastus lateralis during each of the submaximal contractions with a Bagnoli 16‐channel Desktop system (Delsys, Inc., Natick, MA, USA). Prior to detecting EMG signals, the skin over the muscle and patella was shaved and cleansed with rubbing alcohol. Oil, debris, and dead skin cells were also removed with hypoallergenic tape. The sensor was placed over the muscle in accordance with the recommendations described in Zaheer et al. (2012). A reference electrode was placed over the patella. The signals were detected with a surface array EMG sensor (Delsys, Inc., Natick, MA, USA) that consisted of five pin electrodes (Nawab et al., 2010). Four of the five electrodes are arranged in a square, with the fifth electrode in the center of the square and at an equal distance of 3.6 mm from all other electrodes. Pairwise subtraction of the five electrodes was used to derive four single differential EMG channels. These signals were differentially amplified, filtered with a bandwidth of 20 Hz to 450 Hz, and sampled at 20 kHz. Surface EMG signal quality (i.e., signal‐to‐noise ratio >3.0, baseline noise value ≤2.0 µV root‐mean‐square, line interference <1.0) was verified for a 20% MVC assessment prior to data acquisition. A permanent marker was used to outline the borders of the surface EMG sensor following each laboratory visit to ensure consistent placement throughout the study.

Following data acquisition, the four separate filtered EMG signals from the vastus lateralis served as the input to the Precision Decomposition III algorithm, which was utilized via dEMG Analysis software (version 1.1, Delsys, Inc., Natick, MA, USA). Briefly, the algorithm extracts motor unit action potential templates from the EMG data, identifying and separating areas of overlap and assigning specific action potentials to individual motor units. For further information concerning the technical aspects of this algorithm, the reader is directed to the work of De Luca et al. (2006) and Nawab et al. (2010). The surface EMG signals were decomposed into their constituent motor unit action potential (MUAP) trains. We used the Decompose‐Synthesize‐Decompose‐Compare test to remove motor units with detection accuracy below 90.0% (Nawab et al., 2010). For a contraction to be considered, a minimum of five motor units must have been available for analysis. The MUAP trains were then used to calculate a time‐varying firing rate curve for each detected motor unit. All firing rate curves were smoothed with a 1‐second Hanning filter. The mean number of pulses per second (pps) for a 2‐second interval corresponding to the steadiest torque (i.e., lowest coefficient of variation) of each motor unit firing rate curve was calculated. High threshold motor units that were recruited or derecruited during the constant‐torque portion of the contraction were not considered for data analysis. Each motor unit's recruitment threshold was calculated as the relative torque level (% MVC) when the first firing occurred. Contractions in which motor units were detected prior to the onset of muscle torque (i.e., electromechanical delay) were not considered. Similarly, erratic contractions in which the participant decreased torque during the ascending portion of the trapezoid were not analyzed. A custom LabVIEW program (version 8.5, National Instruments, Austin, TX, USA) was utilized to quantify the MUAP peak‐to‐peak amplitude from each of the four channels, with the greatest amplitude value from the four channels being used for subsequent analyses (Contessa et al., 2016).

Once the MUAP amplitude (mV), recruitment threshold (% MVC), and mean firing rate (pps) values for each of the motor units within a contraction were calculated, regression techniques were implemented to examine relationships between these variables. Data were analyzed on an individual participant basis and were neither pooled across multiple contractions nor multiple participants for a given time point. Two approaches were utilized to obtain the motor unit‐dependent variables. First, linear regression was used to identify the linear slope coefficient and y‐intercept of the MUAP amplitude versus recruitment threshold relationship (Pope et al., 2016). Second, an exponential model was applied to each participant's mean firing rate versus MUAP amplitude relationship to yield a *B* term and an *A* term for statistical analysis. With this model, mean firing rate = A*e* ^B^ (MUAP amplitude), where the *A* term is the mean firing rate scale factor, *e* is the natural constant, and the *B* term represents the rate of decay of mean firing rate with increments in MUAP amplitude. Our use of exponential models to characterize the mean firing rate versus MUAP amplitude relationship is consistent with the results of several other studies in this area (Contessa et al., 2016; Miller et al., 2018; Sterczala et al., 2020).

### 2.6 MVC and VA

VA was determined using electrical stimulation of the knee extensor muscles during MVCs. The methodology used in the present study was modeled after that of Park et al. (2008). Stimulation was delivered via two 7.5 cm x 10 cm PALS Neurostimulation adhesive surface electrodes (Axelgaard Manufacturing Co., LTD, Fallbrook, CA, USA), with one over the vastus lateralis and one over the vastus medialis. The electrodes were placed at about one‐third and two‐thirds the distance from the greater trochanter to the superior aspect of the patella (Park et al., 2008; Pietrosimone et al., 2011). When the locations were determined, the areas were shaved with a disposable razor. Prior to applying the electrodes, hypo‐allergenic tape was used to remove oil, debris, and dead skin cells, and the area was cleansed with an isopropyl alcohol pad. After the initial visit to the laboratory, the electrode locations were marked by outlining them with permanent marker to ensure consistent placement on subsequent testing days. An electrical impedance meter (D175 Electrode Impedance Meter, Digitimer Limited, Hertfordshire, UK) was attached to verify that impedance was ≤7 kΩ (Park et al., 2008). When the impedance was >7 kΩ, the electrodes were removed and additional skin preparation was performed. For testing, the electrodes were connected to a constant‐current stimulator (DS7AH, Digitimer Limited, Hertfordshire, UK). Prior to voluntary contractions, the optimal electrical current needed to illicit maximal involuntary torque from the quadriceps femoris muscles was determined by a series of increasing electrically stimulated contractions. Participants were instructed to relax during this process. Stimulations consisted of a paired pulse stimulation, with two 200 µs pulses separated by 10 ms. The first stimulation was set at 120 mA and each successive stimulation increased by 20 mA with ≥20 seconds between stimulations. Optimal stimulation intensity was established when the peak torque elicited resulted in two consecutive decreases. Optimal stimulation intensity was established prior to every testing session.

The interpolated twitch technique was applied to MVCs that lasted approximately 5 seconds. Prior to the test, participants were instructed to push “as hard and fast as possible”. During the test, participants received visual feedback of their torque level on a monitor and strong verbal encouragement. An investigator closely watched the torque‐time curve on a separate monitor. When torque plateaued, a stimulation at the previously identified level was delivered, and the increase in involuntary torque was measured (ITT). Upon feeling the stimulation, participants were instructed to relax. Following the MVC, two more stimulations were delivered at approximately 2 and 4 seconds. The mean of these two values was used to establish electrically evoked torque (EET). VA was calculated as: [1‐ (ITT/EET)] ×100. The attempt with the highest VA for each visit was used for all subsequent analyses.

### 2.7 Concentric strength testing

After MVC and VA testing, maximal concentric strength testing was performed. Concentric testing consisted of four total muscle actions, including two consecutive maximal concentric muscle actions at two different velocities (180ºs^−1^ and 360ºs^−1^). During the tests, participants received strong verbal encouragement. Participants rested for at least 3 minutes between testing of each velocity. The highest concentric peak torque (Nm) value at each velocity served as the dependent variable.

### 2.8 Torque signal processing

The torque (Nm) signal was sampled at 1,926 Hz (a preset commercial hardware device frequency) with EMGworks Acquisition software (version 4.1.7, Delsys Inc., Natick, MA, USA), stored on a personal computer, and processed off‐line with custom written software (LabVIEW 8.5, National Instruments, Austin, TX, USA). The torque signal was filtered using a fourth‐order, zero phase‐shift, low‐pass Butterworth filter with a 10 Hz cutoff frequency. MVC peak torque was defined as the highest mean 500 ms time interval prior to delivery of electrical stimulation. Isokinetic muscle actions were corrected for the effect of gravity on the lower leg in accordance with the procedures described by Aagaard et al. (1995). Concentric peak torque for each muscle action was calculated as the highest 25 ms epoch throughout the range of motion, with the single best muscle action selected for analysis.

### 2.9 Measurement of compliance

Actigraph GT9X accelerometers (ActiGraph Inc, Penscola, FL, USA) were used to ensure participant compliance. Participants were fitted with an accelerometer device on both their right and left ankles, and asked not to remove the devices except during water‐based activities (e.g., showering and bathing). Participants were monitored to determine: (1) their overall wear‐time compliance and (2) compliance with the immobilization protocol. This accelerometer has been validated in previous studies to determine differences in movement patterns between participants’ right and left legs (Dobkin et al., 2011). To meet wear‐time compliance criteria, established by Troiano (2007), participants were required to wear the device for a minimum of 4 days (10 hours per day) over a 7‐day period. As participants were required to wear the device over a 2‐week period, 8 full days of data, including 2 weekend days, were required to meet compliance criteria. To determine compliance, criteria was established based on a previous study by Cook et al. (2006), who examined differences in both the number of steps and intensity of steps between legs in non‐weight bearing participants on crutches.

### 2.10 Statistical analyses

Collectively, this study featured eight dependent variables (MVC peak torque, VA, concentric peak torque at 180°s^−1^ and 360°s^−1^, the linear slope coefficient and y‐intercept of the MUAP amplitude vs. recruitment threshold relationship, and the *A* and *B* terms of the exponential mean firing rate vs. MUAP amplitude relationship). In accordance with recent recommendations (Amrhein et al., 2019), our interpretation of the data was based primarily on effect sizes and 95% confidence intervals for mean differences, and secondarily on null hypothesis significance testing (i.e., *p* values). The eta‐squared statistic, which measures the proportion of variance that can be attributed to a given factor (in this case, time), was examined, with commonly used benchmarks to define small (η^2^ = 0.01), medium (η^2^ = 0.06), and large (η^2^ = 0.14) effects (Cohen, 1988). 95% confidence intervals (CIs) for mean differences and Cohen's *d* effect sizes were also evaluated between time points, with 0.2, 0.5, and 0.8 interpreted as small, medium, and large effects, respectively (Cohen, 1988). Negative *d* values shown throughout the manuscript are indicative of a decrease in the mean value. To examine changes in each variable over time, repeated measures analyses of variance were performed. Greenhouse‐Geisser corrections were implemented when the sphericity assumption was violated. In the event of a statistically significant *F*‐ratio, Bonferroni post‐hoc comparisons were performed to examine differences across time points. An alpha level of *p* ≤ 0.05 was used to reject the null hypothesis. JASP software (version 0.11.1, The JASP Team, 2019) was used for all statistical analyses. Univariate scatterplots displaying individual participant data were created using the templates provided by Weissgerber et al. (2015). The shape and color of each marker represent the same participant across time points and dependent variables.

## 3 RESULTS

The mean ± SD values for each dependent variable at the five time points have been displayed in Table 1. Cohen's *d* effect sizes and mean percent changes comparing each time point to PRE are shown at the bottom of each univariate scatterplot.

**Table 1:** Mean±SD values for each of the dependent variables over time

	PRE	48HR	72HR	1WEEK	2WEEKS
MVC PT (Nm)	189.5 ± 31.9	175.2 ± 37.2	177.7 ± 33.1	169.4 ± 29.4	162.5 ± 29.5
VA (%)	99.7 ± 0.7	91.7 ± 11.2	95.5 ± 4.5	95.0 ± 6.0	92.6 ± 8.4
Concentric PT at 180º⋅s^−1^ (Nm)	85.8 ± 17.7	81.1 ± 19.1	81.4 ± 15.2	77.1 ± 13.5	77.5 ± 17.6
Concentric PT at 360º⋅s^−1^ (Nm)	47.0 ± 9.8	47.1 ± 7.3	49.4 ± 9.4	46.9 ± 8.6	46.2 ± 9.2
APsize vs. RT Y‐Intercept (mV)	−0.0024 ± 0.0023	0.0003 ± 0.0022	0.0006 ± 0.0026	0.0049 ± 0.0019	−0.0053 ± 0.0017
APsize vs. RT Slope (mV ∙ % MVC^−1^)	0.0046 ± 0.0297	0.0049 ± 0.0226	0.0047 ± 0.0356	0.0050 ± 0.0198	0.0056 ± 0.0292
MFR vs. APsize *A*‐term (pps)	23.0 ± 3.6	22.1 ± 2.7	22.1 ± 2.2	22.4 ± 2.3	21.7 ± 2.6
MFR vs. APsize *B*‐term (pps ∙ mV^−1^)	−4.48 ± 1.98	−4.61 ± 2.10	−4.66 ± 1.96	−3.80 ± 1.42	−3.23 ± 1.70

APsize, motor unit action potential amplitude; MFR, mean firing rate; MVC, maximal voluntary contraction; pps, pulses per second; PT, peak torque; RT, recruitment threshold; VA, voluntary activation.

### 3.1 MVC peak torque

**Figure 1:**
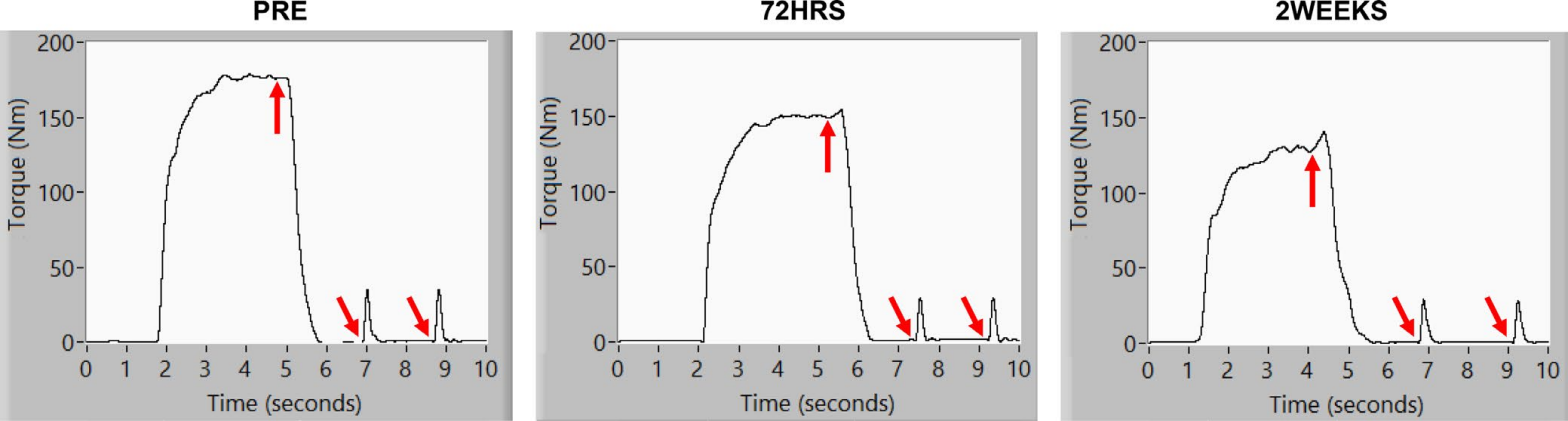
Example LabVIEW waveform graphs from one participant during PRE, 72HRS, and 2WEEKS testing. Each graph shows a torque‐time curve during a maximal voluntary contraction of the knee extensors. Each arrow corresponds to the exact time point when supramaximal electrical stimulation (doublet) was applied to the muscles

**Figure 2:**
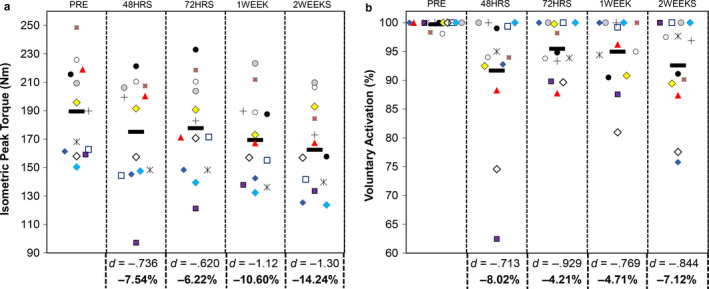
Univariate scatterplots displaying individual participant data for (a) isometric MVC peak torque and (b) voluntary activation across time points. The black, horizontal symbol corresponds to the mean value. Cohen's *d* effect sizes and percentage changes for each time point compared to PRE are shown below the graph

Example isometric torque‐time curves for one participant have been displayed in Figure 1. Individual participant responses for MVC peak torque have been displayed in Figure 2a. The results from the repeated measures ANOVA indicated that there were statistically significant differences in MVC peak torque across time (*F* = 7.025, *p* = .0002) with a moderate effect size (η^2^ = 0.077). The results from the Bonferroni pairwise comparisons indicated that PRE >1WEEK (*t* = −4.04, *p* = 0.0164, *d* = −1.12, 95% CI = −37.1 to −3.04 Nm) and PRE >2 WEEKS (*t* = −4.67, *p* = 0.0054, *d* = −1.30, 95% CI = −46.8 to −7.20 Nm). While no other comparisons demonstrated statistically significant differences, moderate‐to‐large effect sizes between other time points were observed (PRE vs. 48HRS *d* = −.736, 95% CI = −32.9 to 4.2 Nm; PRE vs. 72HRS *d* = −.620, 95% CI = −29.9 to 6.3 Nm; 48HRS vs. 2 Weeks *d* = −.536, 95% CI = −35.1 to 9.8 Nm; 72HRS vs. 2WEEKS *d* = −.675, 95% CI = −36.6 to 6.2 Nm).

### 3.2 VA

Individual participant responses for VA have been displayed in Figure 2b. The results from the repeated measures ANOVA indicated that there were statistically significant differences in VA across time (*F* = 3.490, *p* = 0.0369) with a large effect size (η^2^ = 0.144). The subsequent results from the Bonferroni pairwise comparisons indicated that there were no significant differences among time points (*p* ≥ 0.0578). Nonetheless, as displayed in Figure 2b, moderate‐to‐large decreases in VA were observed at every time point compared to PRE (*d* = −.713 to −.929).

### 3.3 Concentric peak torque

**Figure 3:**
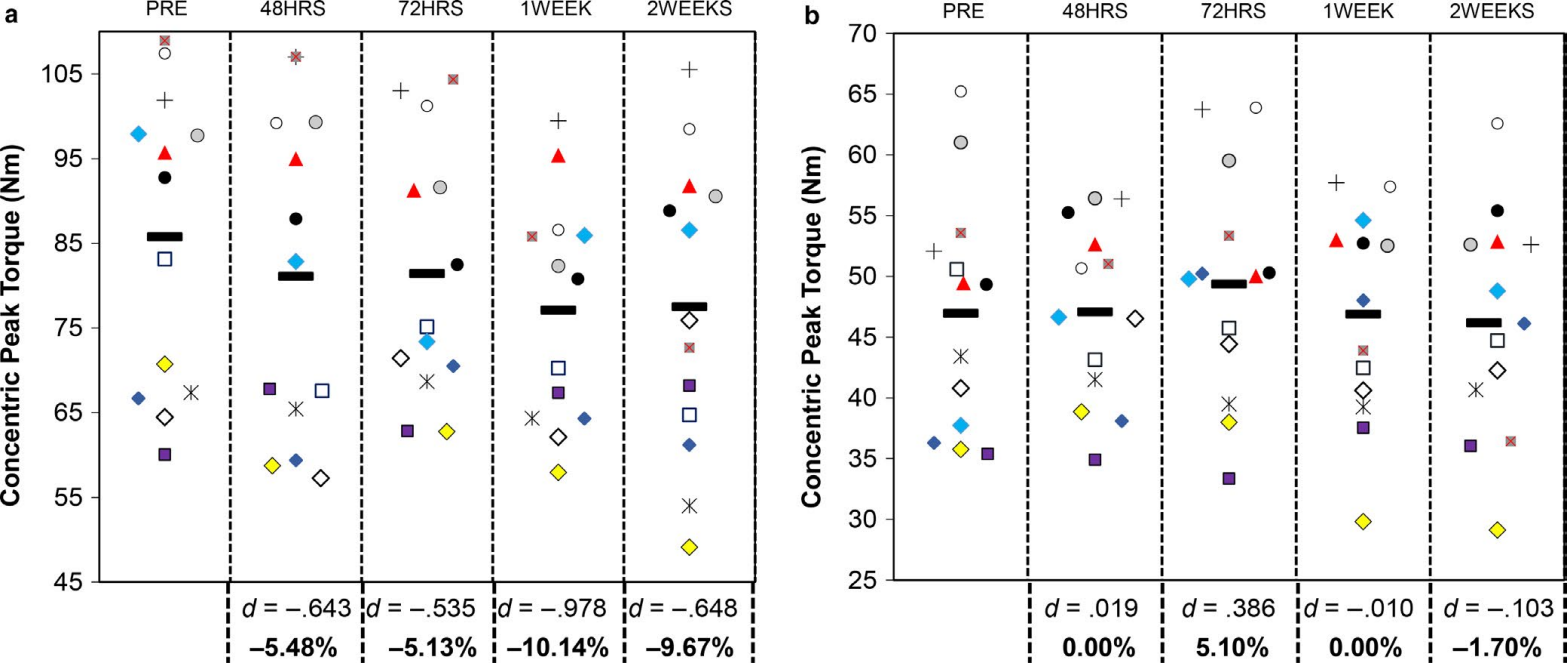
Univariate scatterplots displaying individual participant data for maximal concentric isokinetic strength testing at (a) 180°s^−1^ and (b) 360°s^−1^. The black, horizontal symbol corresponds to the mean value. Cohen's *d* effect sizes and percentage changes for each time point compared to PRE are shown below the graph

Individual participant responses for concentric peak torque have been displayed in Figure 3. The results from the repeated measures ANOVA indicated that there were statistically significant differences in concentric peak torque at a velocity of 180°⋅s^−1^ across time (*F* = 3.525, *p* = 0.0133) with a small effect size (η^2^ = 0.037). The results from the Bonferroni pairwise comparisons indicated that PRE >1 Week (*t* = −3.527, *p* = 0.0417, *d* = −.978, 95% CI = −17.0 to −.2 Nm), but there were no other statistically significant differences despite moderate‐to‐large effect sizes between other time points (PRE vs. 48HRS *d* = −.643, 95% CI = −11.6 to 2.2 Nm; PRE vs. 72HRS *d* = −.535, 95% CI = −12.0 to 3.4 Nm; 72HRS vs. 1WEEK *d* = −.527, 95% CI = −8.7 to 7.9 Nm).

For concentric peak torque at a velocity of 360°s^−1^, the repeated measures ANOVA was not statistically significant (*F* = 0.961, *p* = 0.4376), with a small effect size (η^2^ = 0.0158). The results from the Bonferroni pairwise comparisons indicated that none of the time points were significantly different from each other (*p* ≥ 0.9133), and all effect sizes were *d* ≤ −.509.

### 3.4 Surface EMG signal decomposition output

Given the loss of maximal voluntary strength throughout the course of the study, the mean torque output that the participants needed to reach during plateau of the trapezoidal contractions corresponded to 54.1, 53.3, 55.9, and 58.3% of the PRE MVC at 48HRS, 72HRS, 1WEEK, and 2WEEKS, respectively. In addition, the mean ±SD number of motor units per contraction that the surface EMG signal decomposition algorithm identified with a detection accuracy ≥90.0% was 24 ± 9, 23 ± 7, 21 ± 6, 22 ± 6, and 23 ± 5 at PRE, 48HRS, 72HRS, 1WEEK, and 2WEEKS, respectively. Across all participants and data collection trials, the number of motor units available for analysis ranged from 8 to 37.

### 3.5 MUAP amplitude versus recruitment threshold relationship

**Figure 4:**
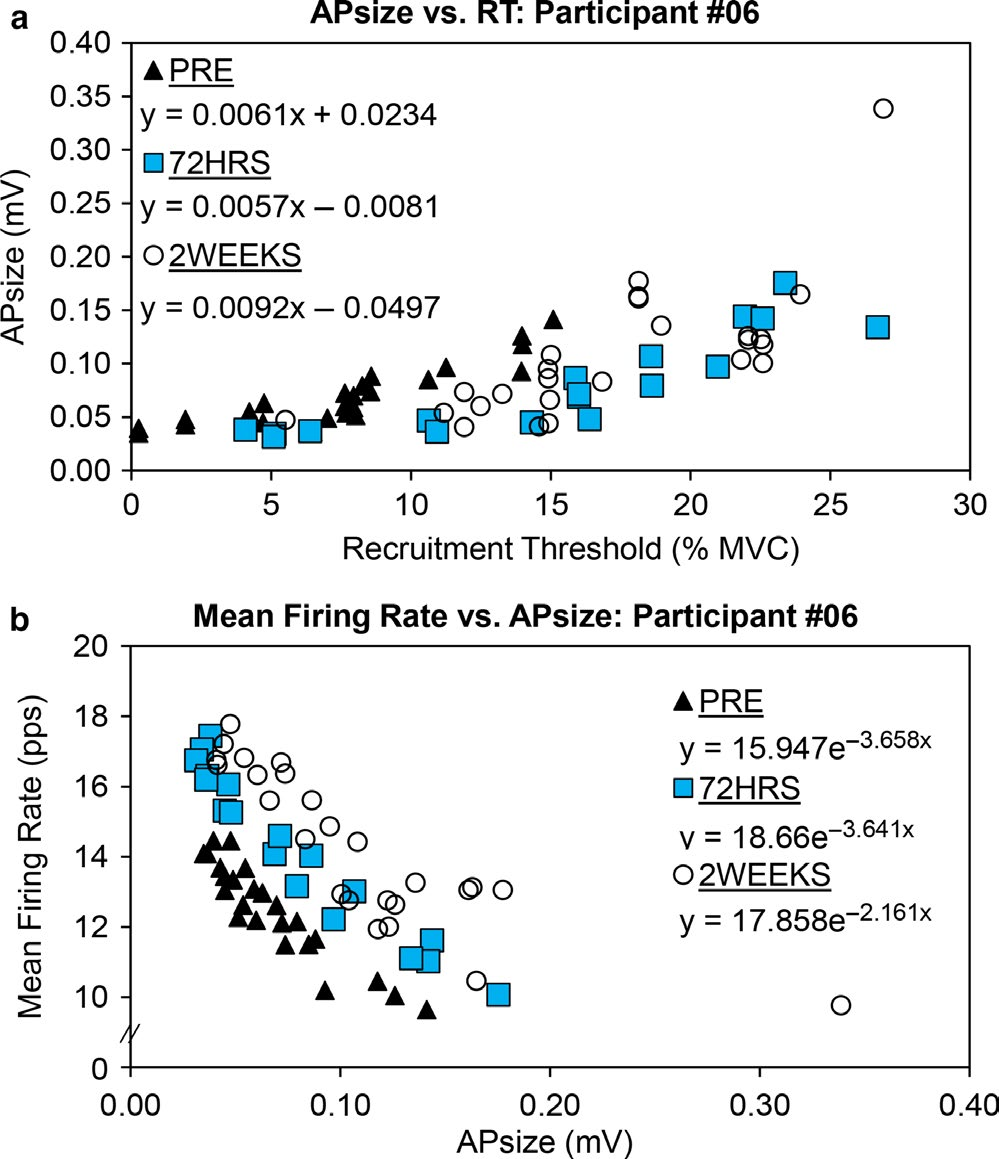
Example motor unit scatterplots for one participant during PRE, 72HRS, and 2WEEKS testing. Each symbol corresponds to a single motor unit. The top graph (a) displays the relationship between motor unit action potential amplitude (y‐axis) and recruitment threshold (x‐axis). The bottom graph (b) displays the relationship between motor unit mean firing rate in pulses per second (y‐axis) and motor unit action potential amplitude (x‐axis). All contractions were performed at 50% of the absolute maximal voluntary contraction peak torque during PRE

**Figure 5:**
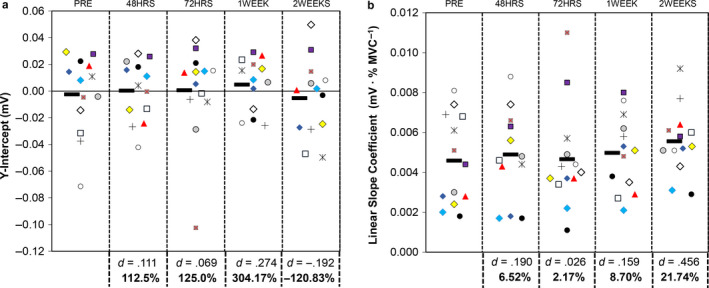
Univariate scatterplots displaying individual participant data for the relationship between the linear motor unit action potential amplitude versus recruitment threshold relationship. The plot to the left (a) shows the y‐intercept, whereas the graph to the right (b) shows the linear slope coefficient. The black, horizontal symbol corresponds to the mean value. Cohen's *d* effect sizes and percentage changes for each time point compared to PRE are shown below the graph

Example motor unit data from one participant has been displayed in Figure 4. Individual participant responses for the linear slope coefficient and y‐intercept of the MUAP amplitude versus recruitment threshold relationship have been displayed in Figure 5. The results from the repeated measures ANOVAs indicated that there were no significant differences among time points for both the y‐intercept (*F* = .279, *p* = 0.8900) and the linear slope coefficient (*F* = .725, *p* = 0.5790). Both variables demonstrated small effect sizes (y‐intercept η^2^ = 0.016; linear slope coefficient η^2^ = 0.027). Each of the y‐intercept pairwise comparisons demonstrated negligible differences between time points (d < .274). For the linear slope coefficient pairwise comparisons, the largest effect size was found for PRE vs. 2WEEKS (*d* = .456, 95% CI = −.0011 to .0030). As shown in Figure 5, these values were highly variable.

### 3.6 Mean firing rate versus MUAP amplitude relationship

**Figure 6:**
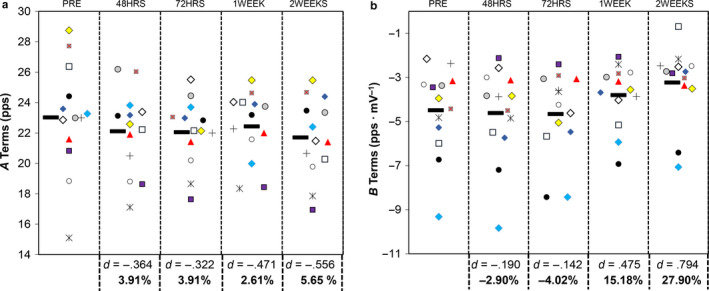
Univariate scatterplots displaying individual participant data for the relationship between the exponential motor unit mean firing rate versus action potential amplitude relationship. The plot to the left (a) shows the A‐terms, whereas the graph to the right (b) shows the B‐terms. The black, horizontal symbol corresponds to the mean value. Cohen's *d* effect sizes and percentage changes for each time point compared to PRE are shown below the graph

Individual participant responses for the *A*‐ and *B*‐terms of the mean firing rate versus motor unit action potential amplitude relationship have been displayed in Figure 6. For the *A*‐terms, the repeated measures ANOVA was not statistically significant (*F* = 1.483, *p* = .2222), with a small effect size (η^2^ = 0.028). The results from the Bonferroni pairwise comparisons indicated that none of the time points were significantly different from each other (*p* ≥ .6830). The only noteworthy effect size was demonstrated for the difference between PRE and 2WEEKS (*d* = −.556).

For the *B*‐terms, the repeated measures ANOVA was statistically significant (*F* = 5.988, *p* = .0005), with a moderate effect size (η^2^ = 0.090). The results from the Bonferroni pairwise comparisons indicated that there were statistically significant differences between 72HRS <1 WEEK (*t* = 3.623, *p* = 0.0344, *d* = 1.007, 95% CI = .048 to 1.676 pps/mV) and 72HRS <2 WEEKS (*t* = 3.553, *p* = 0.0397, *d* = .985, 95% CI = .050 to 2.180 pps/mV). Despite no other statistically significant differences, several other pairwise comparisons demonstrated moderate‐to‐large effect sizes (PRE vs. 1 Week *d* = .476, 95% CI = −.689 to 2.067 pps/mV; PRE vs. 2 Weeks *d* = .794, 95% CI = −.248 to 2.762 pps/mV; 48HRS vs. 1 Week *d* = .591, 95% CI = −.495 to 2.124 pps/mV; 48HRS vs. 2 Weeks *d* = .891, 95% CI = −.093 to 2.858 pps/mV).

## 4 DISCUSSION

The purpose of the present study was to examine the precise time course of muscle strength loss and neuromuscular impairments during short‐term knee joint immobilization in young women. The major findings of this investigation can be highlighted with three main points. First, moderate‐to‐large effect sizes for isometric MVC peak torque were observed within 1 week and continued thereafter, providing evidence that declines in physical function occur rapidly during periods of knee joint immobilization. Given the moderate effect size for the decline in MVC peak torque observed within 48 hours, it is reasonable to suggest that muscle weakness presents as a significant clinical challenge within days of disuse. Second, considerable changes in VA and motor unit behavior were observed, albeit at different time points, which supports an association between immobilization‐induced muscle weakness and neuromuscular impairments. Third, declines in maximal isometric and concentric strength at the two velocities varied among time points, suggesting that the magnitude of immobilization‐induced losses in function may be test specific.

The present investigation adds to a substantial body of evidence showing significant reductions in maximal voluntary strength following periods of disuse, as it is the first study, to our knowledge, to examine the magnitude of maximal voluntary strength loss within a period of only 2 days in young women. While bed rest studies focused on space physiology have been 60 days or longer (Gallagher et al., 2005; Murach et al., 2018), durations between 7 and 30 days have been common in knee joint immobilization or unilateral limb suspension investigations (Campbell et al., 2019), with one lasting 35 days (Tesch et al., 2004). Part of our motivation for carrying out the present study was the similar strength losses observed among investigations between 1 and 2 weeks in duration, suggesting that such changes occur rapidly. As just a few examples, knee joint immobilization studies utilizing a brace model with durations of 7 days have reported strength losses of ~15.0% (Deschenes et al., 2008) and ~13.0% (men) and ~22.0% (women) (Deschenes et al., 2012), whereas others which were twice as long have reported declines of 21.8% (Deschenes et al., 2002) and 22.3% (Oates et al., 2010). Investigations ≥21 days in duration have shown slightly greater strength losses, although work by Cook et al. (2014) demonstrated only a 16.0% decrease in 30 days. Support for rapid decreases in maximal voluntary strength is also supported by work of Hvid et al. (2014), who noted declines of ~9% after only 4 days in older and younger men. When combined with the previous literature, our findings clearly illustrate that meaningful reductions in maximal voluntary strength occur within a period of a few days following knee joint immobilization. In settings where immobilization of the knee joint may be necessary, our results highlight the need to intervene with evidence‐based countermeasures as quickly as clinically appropriate.

Assessment of VA during maximal contractions is frequently used in the literature as a means of examining the ability of the corticospinal tract to recruit motor units to their full capacity and fire them at high rates (Clark & Taylor, 2011). A novel aspect of the present study is that our findings provide support for the hypothesis that reductions in VA accompany strength loss during short‐term knee joint immobilization in young women. While none of the pairwise comparisons were statistically significant (*p* ≥ .0578), moderate‐to‐large effect sizes were demonstrated throughout the intervention. As illustrated in Figure 2b, VA was very high (i.e., >95%) for all participants at baseline. Within 48 hours, however, many of the participants demonstrated decreases in VA, which tended to mirror the decrease in MVC peak torque. It should be noted that decreases in VA during immobilization has not been observed in all studies, and methodological differences could explain these discrepant findings. For example, investigations that have reported decreases in VA during immobilization have been performed for the wrist flexors (Clark et al., 2009, 2014). Studies documenting no change in VA following knee joint immobilization have been performed in young men (Deschenes et al., 2017; Hvid et al., 2018). We should also note that while the assessment of VA has value in neuromuscular research (Clark & Taylor, 2011; Herbert & Gandevia, 1999), we are unable to state with certainty the specific structures throughout the corticospinal tract which were affected by knee joint immobilization. Larger studies employing more advanced neurophysiological techniques which enroll both men and women are needed to confirm the gender‐specific factors related to declines in VA during short‐term knee joint immobilization.

Although a variety of neuromuscular tools have been used to quantify mechanistic changes following disuse (Campbell et al., 2019; Clark et al., 2009, 2014; Cook et al., 2010, 2014; Deschenes et al., 2017), these studies did not specifically examine motor unit recruitment or firing rate behavior during voluntary contractions. As such, the present study provides unique insight into the motor unit adaptations that occur during short‐term knee joint immobilization in college‐aged women. The most important finding was that there were no significant changes in the slope or y‐intercept for the MUAP amplitude versus recruitment threshold relationship, whereas the mean firing rate versus MUAP amplitude relationship was affected during the later stages of the protocol. Given that the peak‐to‐peak amplitude of the MUAP waveform reflects the size of the motoneuron (Olson et al., 1968) and its constituent muscle fibers (Henneman & Olson, 1965; Henneman et al., 1965), assessment of the relationship between MUAP amplitude and recruitment threshold has proven to be a useful analytical tool for studying adaptations to resistance training (Pope et al., 2016; Sterczala et al., 2020). By utilizing B‐mode ultrasonography to examine skeletal muscle hypertrophy, Pope et al. (2016) concluded that the increase in the slope coefficient of the MUAP amplitude versus recruitment threshold relationship was closely related to the increase in vastus lateralis cross‐sectional area, thereby providing support for using the techniques presented herein as a non‐invasive measure of motor unit plasticity. Similar findings were recently reported by Sterczala et al. (2020). Whereas the present results indicated that knee joint immobilization did not alter this relationship, shifts in the *B*‐term of the mean firing rate versus MUAP amplitude relationship were evident. Overall, this would suggest that changes in firing rate, rather that motor unit size, may be more sensitive to detecting disuse‐related adaptations. While the lack of previous disuse or immobilization studies which have studied motor unit control makes data interpretation difficult, we speculate that our findings can be explained by a decrease in motor unit firing efficiency as the knee extensor muscles progressively weakened. The “efficiency of electrical activity” was initially described by DeVries (1968) and later expanded upon by Moritani and deVries (1979). The foundational premise of the efficiency of electrical activity technique is that changes in the level of motoneuron excitation reflect the demand on the central nervous system necessary to produce a given amount of force or torque. Increases in the level of surface EMG amplitude needed to produce a given absolute force level, in turn, reflect less efficiency (DeVries, 1968). While additional studies are needed to confirm these findings, a decline in the neuromuscular system's firing efficiency may be a plausible explanation for the results of the present study.

It is important to reiterate that the absolute torque levels at which bipolar surface EMG signals were acquired and subsequently decomposed remained consistent throughout the study. As such, when participants demonstrated reductions in MVC peak torque, a greater relative percentage of their new maximal torque was necessary to perform the trapezoidal isometric contractions. We believe that this is a crucial consideration that is often inadequately considered in the literature, and would suggest that our findings may not be applicable to scenarios in which only relative percentages of the MVC are used throughout the disuse period. Although preceding studies have not specifically examined disuse, some investigators who have utilized a constant, absolute force/torque level have been able to detect acute or chronic motor unit changes (Pope et al., 2016; Stock et al., 2012), whereas no changes have been reported when only relative levels were examined (Beck et al., 2011).

To our knowledge, this study was the first to examine short‐term (≤ 1 week) knee joint immobilization in women alone. Although literature is limited, it appears that there are gender differences in response to knee joint immobilization, with women showing greater declines in isometric strength. The most relevant study to the present investigation was conducted by Deschenes, McCoy, Holdren, et al. (2009). These authors (Deschenes, McCoy, Holdren, et al., 2009) examined differences in muscle strength loss and neuromuscular impairments following 1 week of knee joint immobilization in 10 men versus 10 women. While both genders showed significant changes in MVC peak torque, the change was nearly twice as great for the women. Differences between men and women might also be noted during recovery from immobilization. Clark et al. (2009) studied gender differences in the loss of strength following 3 weeks of wrist immobilization, as well as subsequent recovery. The authors (Clark et al., 2009) reported that men and women lost voluntary strength at a similar rate during immobilization. Following 1 week of recovery, however, voluntary strength had returned to baseline in the men, but remained approximately 30% less than baseline in the women. Overall, much more research is needed to determine the optimal post‐knee joint immobilization rehabilitation stimulus for men versus women.

Our exploration of knee joint immobilization‐induced weakness at velocities of 180°⋅s^−1^ and 360°⋅s^−1^ is also novel. This is the fastest velocity at which peak torque has been shown to decline during short‐term disuse in women. Previously, two studies have reported declines in women at velocities of ~120°s^−1^ (Deschenes, McCoy, Holdren, et al., 2009; Deschenes et al., 2012), while one study has reported similar decreases in men (Deschenes et al., 2009). Our findings of decreases in concentric peak torque at 180°⋅s^−1^ suggest women may experience losses of knee extensor strength throughout a broader range of velocities than previously known. At velocities of 360°⋅s^−1^, decreases in strength have not been reported for either gender. The fact that the time course and magnitude of decreases in peak torque differed for maximal isometric and concentric tests at two velocities have important implications for designing and implementing assessments in clinical settings. Specifically, our findings highlight the fact that, while dynamic strength tests may offer greater external validity, rapid velocities may not be sensitive enough to identify impairments in function otherwise observed with isometric strength tests. Based on both our findings and the previous literature (Deschenes, McCoy, Davis, et al., 2009; Deschenes, McCoy, Holdren, et al., 2009; Deschenes et al., 2012), we recommend that clinicians seeking to evaluate knee joint strength during rehabilitation utilize maximal isometric muscle actions or concentric muscle actions performed at velocities ≤180°s^−1^.

Like all studies, the present investigation had limitations. Three seem particularly pertinent. First, a concern when designing this study was whether the data collection trials would serve as a training stimulus, thereby preventing the loss in muscle strength otherwise observed during immobilization. As such, we minimized the number of strength tests and submaximal contractions that the participants performed. Support for the notion that testing protocols may serve as a training stimulus was well exemplified by the work of Mattocks et al. (2017), who reported similar improvements in one repetition maximum strength for participants who went through a progressive resistance training program versus simply practicing the test. While implementation of additional testing sessions may have prevented some of the loss in strength, we observed no significant differences and small effect sizes in MVC and concentric peak torque between 1 Week and 2 Weeks. In fact, the declines in strength from PRE to 1 week were substantially greater than that observed between 1 Week and 2 Weeks, despite the assessments at 48 h and 72 h. This gives us confidence that if the 48 h and 72 h assessments prevented further strength loss, the effect was not detected. Second, our study did not include analysis of the non‐immobilized limb as a control. While this may be perceived as a limitation, our concern when designing the study was that mobility with an immobilized limb would serve as a training stimulus for the non‐immobilized limb, similar to that observed during short‐term cross‐education investigations (Carr et al., 2019). Third, it should be noted that many of the declines in muscle strength between later time points were not statistically significant despite moderate‐to‐large effect sizes. While this suggests that the present study may have been underpowered, it should be highlighted that our final sample size of 13 seems greater than many of the previous studies in this area. It is our hope that our inclusion of effect sizes will help future investigators in the design of larger, more definitive short‐term knee joint immobilization trials. As a final note, we should acknowledge that some of the responses to knee joint immobilization varied among participants and time points. Figure 2 highlights this interindividual response heterogeneity, with one participant showing a dramatic decline in MVC peak torque and VA at 48HRS, but gradually returning to baseline. Close examination of these plots shows examples of situations where negligible changes or even slight improvements in strength or neuromuscular function were observed. Rather than shying away from these unique responses, we believe that understanding the biological, mechanistic, and lifestyle factors which determine why some individuals are able to maintain their functional status during periods of disuse, while others are dramatically affected, requires additional consideration from investigators.

## 5 CONCLUSIONS

In young women, immobilization‐induced decreases in knee extensor muscle strength occur rapidly. Based on the VA and motor unit behavior results, it appears that the decline in function is mediated by impairments in the central nervous system's ability to activate motor units, although the time course between changes may be variable. The differences in strength responses among the three velocities tested has implications for designing strength testing protocols in clinics and rehabilitative settings, with isometric muscle actions likely showing the greatest change in strength loss. From a clinical perspective, these findings highlight the need for early interventions that may mitigate immobilization‐induced declines in muscle function. Larger clinical trials including both men and women are needed to determine if gender‐specific prevention or rehabilitation protocols are necessary.

## CONFLICT OF INTEREST

All authors declare no competing interests.

## Data Availability

The data that support the findings of this study are available from the corresponding author upon reasonable request.
